# In this issue: Volume 115, Issue 12, December 2024

**DOI:** 10.1111/cas.16411

**Published:** 2024-12-02

## Usefulness of multigene liquid biopsy of bile for identifying driver genes of biliary duct cancers

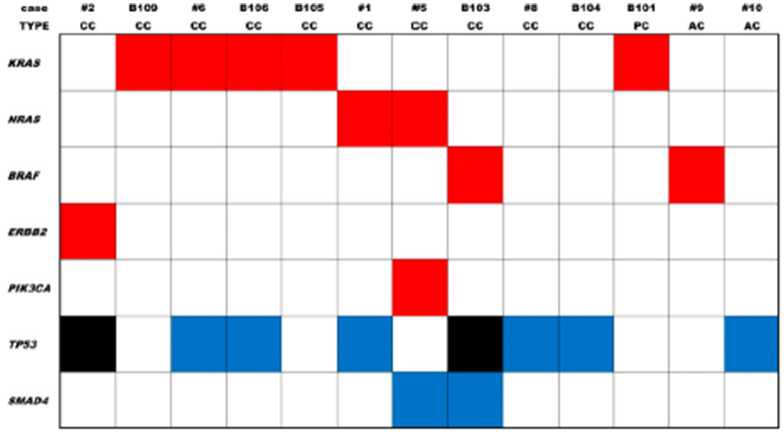

Biliary tract cancers (BTCs), which include cholangiocarcinoma (CC) and ampullary carcinoma, are often diagnosed at advanced stages, resulting in limited treatment options and poor survival rates. To improve outcomes, there is a pressing need for diagnostic methods that detect BTCs earlier. Liquid biopsy (LB) offers a promising approach. This minimally invasive technique analyzes cell‐free DNA (cfDNA)—fragments of DNA shed from dead cells, including cancer cells—found in body fluids like blood or bile. Analyzing cfDNA can provide genetic insights into cancer development, making LB a useful tool for BTC diagnosis.

In this study, Ito and Ando et al. investigated bile as a source for cfDNA in BTC diagnosis, given that bile contains significantly higher levels of free nucleic acids than blood plasma and can be collected through endoscopy. The researchers used a multigene panel LB system to analyze cfDNA from bile samples of 24 patients with BTC, which included 17 cases of CC, 3 cases of ampullary carcinoma, 2 cases of pancreatic cancer, 1 case of intraductal papillary mucinous carcinoma, and 1 case of insulinoma. Plasma cfDNA samples were also collected from 17 of these patients to compare the effectiveness of bile and plasma in identifying mutations. Bioinformatics methods were then used to analyze the genetic alterations in both sample types.

The analysis found that cfDNA levels in bile were on average 68.2 times higher than in plasma, underscoring bile as a more sensitive medium for mutation detection in BTC. *KRAS*, which encodes a protein involved in cell proliferation and death, was the most frequently mutated gene. Among the 17 CC cases, at least one driver mutation was detected in 10 cases. The driver mutations in the bile cfDNAs were detected in 13 CC cases (54%), whereas those in the plasma cfDNAs were detected in only 4 CC cases (17%).

These findings suggest that bile‐based LB can more reliably detect BTC‐related mutations, offering a valuable tool for early diagnosis and tailored treatment strategies. Such advances could improve patient outcomes by enabling BTC detection at earlier stages, expanding treatment options and potentially increasing survival rates.

https://onlinelibrary.wiley.com/doi/full/10.1111/cas.16365

## The novel and potent CD40 agonist KHK2840 augments the antitumor efficacy of anti‐PD‐1 antibody and paclitaxel

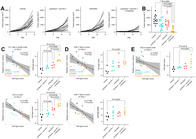

Immune checkpoint inhibitors (ICIs) can be potent anticancer agents that function by blocking specific proteins. These proteins usually prevent the immune system from attacking healthy cells. But cancer cells often use checkpoints to hide from the immune system, which facilitates their progression without being attacked. ICIs block these checkpoints, thereby allowing the immune system to detect and eliminate cancer cells. However, many patients show inadequate response to treatment with ICIs alone, highlighting the need for improved approaches.

A major reason associated with the inadequate response seen with ICI treatment is the lack of tumor‐reactive T‐cells. T‐cells are a type of immune cells that are essential for targeting and eliminating tumor cells. Chemotherapeutic drugs are sometimes used with ICIs to increase T‐cell count and improve the immune response to cancer, but this approach does not yield positive results in all patients.

Researchers are thus investigating another type of drug called CD40 agonists, which can boost the immune system by activating a pathway that increases T‐cells. To add to these ongoing efforts, Kobayashi et al. have developed a novel, engineered CD40 agonist, KHK2840. KHK2840 is a specially engineered antibody designed to activate the immune system against cancer more effectively than hitherto reported CD40 agonists.

In Kobayashi et al.'s study using human CD40 transgenic mouse models, KHK2840 showed antitumor activity and reduced tumor growth significantly on its own. When the researchers tested its effects in combination with anti‐programmed cell death 1 (anti‐PD‐1), a common type of ICI, and paclitaxel, a chemotherapeutic drug, they observed a synergistic effect that led to an appreciable reduction in tumor growth and complete elimination of the tumor in some cases.

Safety is a crucial aspect of any new cancer treatment. Accordingly, Kobayashi et al. conducted toxicity tests on monkeys in which KHK2840 was found to induce CD40 signaling with some adverse effects but no mortality. Other CD40 agonists investigated in clinical trials have not performed as expected, making KHK2840 a promising alternative.

These findings suggest that KHK2840 is a potent new drug in cancer immunotherapy, particularly when used in combination with ICIs and chemotherapy to achieve a stronger, more sustained immune response against cancer.

https://onlinelibrary.wiley.com/doi/full/10.1111/CAS.16366

## Loss of USP10 promotes hepatocellular carcinoma proliferation by regulating the serine synthesis pathway through inhibition of LKB1 activity

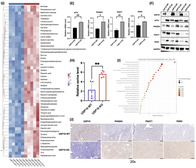

Liver cancer is one of the most common and lethal kinds of cancer worldwide. The liver plays a crucial role in managing proteins, sugars, and fats—molecules essential for cell growth and replication. When liver cancer cells alter the liver's metabolism, they access these resources, allowing them to grow rapidly. One important metabolic pathway that cancer cells often change is the serine synthesis pathway (SSP). When this pathway is out of balance, it harms the liver in two ways. First, cancer cells can generate large amounts of serine, a key amino acid required for building proteins, DNA, RNA, and even cell membranes, all of which are needed for cell division. Second, SSP uses glucose, leading healthy liver cells to release stored sugars and convert fats into sugars, which the cancer cells then consume, creating a harmful cycle.

In this study, Ma et al. looked at the role of an enzyme called ubiquitin‐specific protease 10 (USP10) in regulating the SSP of hepatocellular carcinoma (HCC) cells. They found strong ties between SSP and USP10 in HCC cells. First, HCC cells with non‐functional SSP genes also had lower levels of USP10. Second, HCC cells with USP10 mutations had higher levels of serine and grew faster. Patients with low levels of USP10 had more aggressive tumors, a shorter period of remission before cancer re‐emerged, and lower survival rates.

The team also found that USP10 regulates SSP by activating a tumor‐suppressing protein called LKB1. If activated LKB1 can be produced in the cancer cell, SSP can be suppressed even without USP10.

Overall, these findings suggest that USP10 plays a crucial role in the growth of HCC and could be an important factor in both diagnosis and treatment. By monitoring USP10 levels, doctors might gain valuable insights into how aggressive a patient's cancer could be, which could guide treatment decisions. Furthermore, boosting USP10 activity may slow down cancer progression, offering hope for improved survival rates. Ultimately, targeting USP10 could lead to new strategies in managing liver cancer, providing patients with better options for their care.

https://onlinelibrary.wiley.com/doi/full/10.1111/cas.16336

